# Epithelioma

**Published:** 1884-12-20

**Authors:** H. M. Lawson

**Affiliations:** Cuthbert, Georgia


### Southern Medical Record.

EDITORS:

THOMAS S. POWELL, M. D. R. C. WORD, M. D.

*R. C. WORD, M.D., Managing Editor.*

☞All Communications and Letters on Business connected with the RECORD must be addressed to the Managing Editor.

VOL. XIV. ATLANTA, GA., DECEMBER 20, 1884. No. 12.

### ORIGINAL AND SELECTED ARTICLES.

### EPITHELIOMA.

By H. M. LAWSON, M. D., Cuthbert, Georgia.

The following case is confirmatory of what I have already published concerning the use of arsenic in the treatment of malignant and allied affections. (See SOUTHERN MEDICAL RECORD for April, 1884.)

Mr. S. applied to me early in June last for relief from ephithelioma. He was forty-five years of age ; by occupation, a carpenter ; of good habits, but of cancerous parentage, his mother having died of that disease.

The disease, which was located on the left temple, had existed eight years. He had been under the treatment of five or six cancer doctors ; indeed, from his account, he seemed to have traveled over the country in search of them, and no sooner exhausted the resources of one than he would set out in search of another. Various local applications were made during this time, but without benefit.

I put him, at the commencement, upon one-fourth of a grain of arsenious acid ; but this dose failing to prove laxative, it was gradually increased. But this was not properly done, because the patient was too far from me, and the prompt action on the bowels—which indicates that the full tonic and alterative action of the remedy is being brought to bear upon the patient, also safety from its cumulative effects—was neither induced nor maintained as it should have been ; and, although three-fourths of a grain was finally reached, properly aided by rhubarb, the bowels did not act in a satisfactory manner.

Notwithstanding these disadvantages, the remedy was continued nearly four weeks ; then a caustic application was made to the part, and, after the separation of the slough, the parts were healed in three weeks ; and there is no indication of a return of the disease up to this time.

The caustic application used in this case, if not new to the profession, is seldom used. Its source and mode of application is as follows :

A quantity of the leaves and stems of the *Rumex Acetocella* is gathered, washed, and subjected to pressure. The juice thus obtained is exposed to the sun and air in a shallow vessel, as a plate, until it acquires the consistence of an ordinary vegetable extract, then spread as a plaster, and applied to the part.

In this case the slough was detached in three days.

The treatment of this case convinced me fully of the necessity of having such patients in close proximity to the medical attendant; otherwise, it is almost impossible to conduct the treatment (especially the treatment by arsenic) properly. A hospital devoted to the purpose affords the best opportunity for any treatment, and is indispensable for that herein recommended ; and I am glad to know that such a hospital is in course of erection, where this so long neglected class of sufferers will receive the treatment their condition so imperatively demands.

In the case under consideration the patient attributed the cure to the caustic application. Persons who know nothing of the effects of remedies, always attribute the cure to the remedy last employed. Such, at least, is my experience ; but those who are acquainted with cancerous affections are aware that caustic agents alone are of little avail. My own experience is, that no caustic agent whatever would have proved of any value in the case, had it not been preceded by an arsenical course of sufficient length to have subverted the morbid action ; after which one caustic agent, properly used, would have proved as efficient as another.

I have this reason for thinking so : I treated a patient several years ago who, after a properly conducted arsenical course, was relieved in like manner, as was the one under consideration, by an application of the chloride of zinc. This last mentioned case was true cancer. Both of these patients had been previously treated by caustics alone, but without relief; both were cured by preceding the local treatment by a course of arsenic.

I am not sure that the rumex extract was used in either of these cases previously to their coming under my treatment, but believe it was in both, because the parties who had treated them relied chiefly on it, or on a similar extract prepared from the *Phitolacca decandra.*

The manner in which I use arsenic in malignant diseases will be found in the number of the SOUTHERN MEDICAL RECORD already referred to.

I hope that the members of the profession, who have opportunity, will repeat my experiments, and report results through the medical journals; and that those having such cases, but without the time or opportunity of treating them, will put them in commucation with me by letter. I have room for three or four cases in my own house, where I can conduct the treatment as I prefer and intend to do in the future—that is, to give the medicine with my own hand.

There may be a few members of the profession who entertain the views, about cancer and arsenic, expressed by my old friend Dr. B., who expressed himself as follows: "The medical profession has long since declared that cancer is incurable, also that arsenic is poisonous; therefore I do not wish to have anything to do with the former, and less to do with the latter." It is needless to say that, from such practioners as this the profession has little or nothing in the way of advancement to expect.

Of the two vegetable extracts mentioned in the article, I have not experience sufficient to decide as to whether they will prove of value in the treatment of malignant disease, or not, but believe that both possess valuable properties, especially the rumex; and I intend to test them fully, as soon as suitable cases afford me the opportunity.

I believe in the curability of malignant and allied diseases. I further believe that but few things are denied to properly and persistently directed endeavor. My investigations in this direction have been attended by results so encouraging that I shall continue them. It is immaterial to me whence comes the remedy—whether from the animal, vegetable, or mineral kingdoms, or from all of them. I shall utilize everything that will aid me in my self-imposed task, and respectfully ask the aid of the profession, so far as to enable me to obtain cases upon which to continue my investigations; and since they, as well as suffering humanity, are to be partakers of the benefits of my labors, I feel assured that I will not ask in vain.

